# Palliative Care Coverage across European National Health Systems: Proposal of a Synthetic Indicator

**DOI:** 10.3390/ijerph182010753

**Published:** 2021-10-13

**Authors:** Miguel Antonio Sánchez-Cárdenas, Eduardo Garralda, Edgar Benítez, Natalia Arias-Casais, Danny van Steijn, Carlos Centeno

**Affiliations:** 1 ATLANTES Global Palliative Care Observatory, Institute for Culture and Society, University of Navarra, 31001 Pamplona, Spain; egarralda@unav.es (E.G.); narias@unav.es (N.A.-C.); dvansteijn@unav.es (D.v.S.); ccenteno@unav.es (C.C.); 2 IdiSNA—Instituto de Investigacion Sanitaria de Navarra, 31001 Pamplona, Spain; 3 DATAI, Instituto de Ciencia de los Datos e Inteligencia Artificial, University of Navarra, 31001 Pamplona, Spain; ebenitezs@unav.es

**Keywords:** palliative care, integrated, healthcare indicators, Europe, national-level

## Abstract

Background: The coverage of palliative care (PC) may be understood as a country’s capacity to offer prevention and relief from serious health-related suffering in relation to an existing need. The aim of this study is to estimate European countries´ coverage capacities. Method: Secondary analysis of three indicators, including the number of specialized services (SSPC), integration capacity scores (ICS) and the PC needs. By means of a K-medians clustering supervised algorithm, three coverage profiles were obtained: (1) Advanced: countries with high ICS and SSPC, and low PC needs; (2) Limited: countries with low ICS and SSPC, and low PC needs; and (3) Low: countries with low ICS and SSPC and high PC needs. Results: On average, the ratio of specialized services per population was 0.79 per 100,000 inhabitants, the average ICS was 19.62 and the average number of deceased patients with SHS per 100,000 inhabitants was 5.69. Twenty countries (41%) reached an advanced coverage profile. Nine countries (18%) demonstrated a limited coverage profile; and 20 countries (41%) fell under a low-coverage capacity. Conclusion: The level of palliative care coverage across Europe shows that 59% of European countries have either limited or very low availability of PC resources as regards their palliative care needs.

## 1. Introduction

Palliative care development aims to ensure access by all children and adults experiencing serious health-related suffering to timely and effective palliative care [1].The palliative care development in Europe has been studied, using indicators to evaluate the public health components involved in the palliative care activity and the integration into other levels of health care system [2]. Diverse studies and methods over the last decade have aimed at evaluating the degree of PC development on a country-by-country basis [3]. Initially, national development was measured using exclusively the morphine consumption indicator, while the first two editions of the most cited study used experts’ global qualitative estimates as a way of comparing their perception of PC in their own countries with standard descriptions of different levels of development [4,5]. A similar approach, combining quantitative data with qualitative estimations, was used in the QofDeath Index [6]. Later on, the evaluation of development started to build on indicators according to the dimensions of the WHO Public Health Strategy for the Integration of Palliative Care: appropriate policies, use of medicines, education and adequate service provision [7]. Commonly, a number of studies focused on measuring specialized palliative care on a regional basis through experts’ knowledge: Europe, Latin America, Africa and the Eastern Mediterranean [1,8,9,10,11]. Others relied on official sources, such as the Country Capacity Survey, conducted by the WHO [12]. One of the main indicators used was the number of specialized palliative care services, as it represents a directly-related measure of access to care [13].

However, from a public health perspective, palliative care provision does not rely only on specialized services (composed of professionals with specific training in palliative care), but also on any resources, specialized or non-specialized, that are integrated into the health system, pursuing the relief of suffering and symptom control for patients in need. This means access at all levels of care, independently of the providers and care setting, the age, or the disease of the patient [14]. Therefore, indicators examining palliative care provision at the primary care level, provided by non-PC specialists, outside the specialized settings, for child populations, and diseases other than cancer, are also needed to understand coverage comprehensively. These indicators need to complement the widely accepted indicator on the number of specialized palliative care services per population. In this sense, a recent publication developed a first estimation of the capacity of countries to provide integrated palliative care [2].

To date, the absence of a global indicator for palliative care coverage has been reported as an important barrier to PC inclusion in global efforts towards universal health coverage [15]. The synthesis of primary indicators of PC coverage is possible with the use of statistical techniques used in other areas of public health, such as the epidemiological surveillance of chronic non-communicable diseases. These have been shown to successfully provide information for improving decision-making in national health systems. The aim of this work is to propose a first synthetic measure that compares both specialized and non-specialized palliative care provision with the country´s palliative care needs, as a way of analyzing the level of PC coverage in the WHO European Region.

## 2. Materials and Methods

The coverage of palliative care is understood as the capacity of countries to offer prevention of and relief for serious health-related suffering, in relation to an existing need, with an adequate balance between specialized palliative care services and other resources available in the health system [2]. A statistical approach to evaluate palliative care coverage in the national health systems country-by-country across Europe was applied following the Guidelines for Accurate and Transparent Health Estimates Reporting (GATHER) [16].

### 2.1. Primary Indicators and Data

Data on specialized palliative care services, resources in other areas of the health system, and the burden of the need for PC in the population for 51 countries, all from the EAPC Atlas of Palliative Care in Europe 2019, were used [17]:

1. **Specialized palliative care services (SSPC)** refers to the total number of reported specialized services per 100,000 inhabitants [13].
2. **Resources in other areas of the health system: Integration Capacity Score (ICS)** refers to an indicator synthesizing PC activity in pediatrics, cardiology, oncology, primary care, long-term care, and volunteering. This score has a maximum value of 53 points and allows the classification of countries at a high level (score from 53–23), middle level (score from 22–11), and low level (<10 points) [2].
3. **The standardized need for palliative care for each country** refers to the total number of patients who died due to SHS per 100,000 inhabitants in 2017. The burden of serious health-related suffering was based on the Lancet Commission report [18], using base mortality rates for 17 conditions from the WHO World European Mortality Database (EMD) [19].

The data for each indicator were independently consolidated in a data analysis matrix and processed in the statistical package R, version 4.1.1. Countries in which it was not possible to obtain information on any of these three indicators were excluded from the analysis.

### 2.2. Synthetic Coverage Indicator

The indicator of specialized PC services per 100,000 inhabitants and the ICS indicator were evaluated against the indicator of the need for PC. Through unsupervised clustering techniques of k-means to these variables, a cluster of coverage profiles was elaborated. This algorithm sought to classify countries into k groups, in which each observation belonged to the cluster with the closest mean. This was achieved through the minimization of the following objective function:

$$J=\overset{k}{\underset{j=1}{\sum }}\overset{n}{\underset{i=1}{\sum }}||x_{i}^{\left(j\right)}-c_{j}||{}^{2}$$

where, $x_{i}^{\left(j\right)}$ is each country, $c_{j}$ is the center of the cluster j, n is the total number of countries, k is the number of clusters, and $\left||x_{i}^{\left(j\right)}-c_{j}|\right|$ is the distance between the point $x_{i}^{\left(j\right)}$ and cluster center $c_{j}$. Unlike supervised techniques, clustering is guided by the closeness of the data without the intervention of the investigator. The only restriction is imposed by the number of “k-neighborhoods” for which the closeness of the variables must be found.

Using this formula, three possible coverage profiles were identified: (1) Advanced: countries with a high score on ICS and SSPC and low PC needs; (2) Limited: countries with low levels of need, and low scores on ICS and SSPC; and (3) Low: countries with high levels of need and low levels of ICS and SSPC. This classification was denominated the Country PC Coverage Level (CCL).

### 2.3. External Validation

Since no gold-standard measures of palliative care coverage exists, the world map of palliative care categories, where specialized and integrated provision converge, was used to trial the Country PC Coverage Level [20].

### 2.4. Patient and Public Involvement Statement

No patients or public were involved in the design, the recruitment or any other stages of the study. The development of the research question and the outcome measures were based mainly on macro-level data regarding the coverage of palliative care to health systems.

## 3. Results

Available information for the three indicators was identified in 49 countries. The data for Liechtenstein and Monaco were not available. The average of specialized services was 0.7 ± 0.6 per 100,000 inhabitants, with 18 countries reporting a ratio above 1. Austria, Ireland and Luxembourg demonstrated the highest ratio of services per capita. The Integration Capacity Score measuring the available palliative care resources for children, for patients of all ages, at the primary care level, for oncology and cardiac patients in the health system classified 17 countries with high degrees of PC integration, 18 countries with medium integration, and 16 with low integration. The Netherlands, the United Kingdom, Germany, Switzerland, and Belgium presented better figures in the integration of palliative care in different areas of their health systems. The specific prevalence of deaths due to SHS per 100,000 inhabitants showed an average of 5.6 ± 1.9. In some countries, palliative care needs were over 8.0 people per 100,000 inhabitants: Romania, Serbia, Latvia, Montenegro, and Ukraine (Table 1).

### 3.1. Country Coverage Level (CCL)

**Figure 1:**
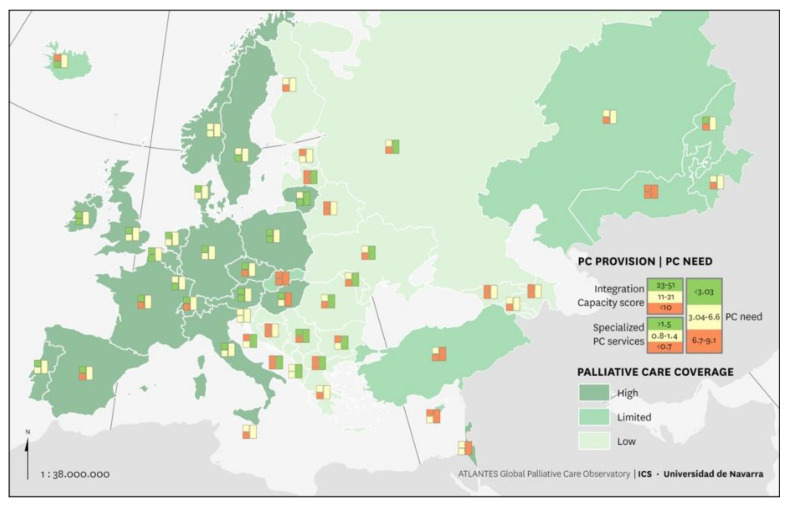
Palliative care country coverage level in Europe.

The CCL classified countries in three levels of coverage (Table 2). Twenty countries (41%) reached an advanced coverage profile (SSPC average, 1.27; ICS 31.45; and 5.32 deceased patients per 100,000 inhabitants). The highest values of the synthetic indicator were recorded in The Netherlands, the United Kingdom, and Germany. Nine countries (18%) demonstrated a limited coverage profile (SSPC average 0.38, ICS 11.67, and 3.18 deceased patients per 100,000 inhabitants); and 20 countries (41%) demonstrated a low coverage capacity (SSPC average 0.50, ICS 12.10, and 7.19 deceased patients per 100,000 inhabitants) (Figure 1).

Countries with limited and low coverage levels showed similar scores for the SSPC and the ICS, but differed in the number of people needing palliative care.

### 3.2. External Validation

As compared with the World Map of Palliative Care 2019 levels [20], 19/20 (95%) of the countries with an advanced coverage profile corresponded either with advanced (75%) or preliminary (20%) integration categories. A majority of the countries with limited coverage profile demonstrated the generalized provision levels (5/9, 56%), while countries with low coverage profiles corresponded mostly with isolated or generalized provision levels (14/20, 70%). Countries at this last level presented an uneven distribution regarding World Map levels (Table 3).

## 4. Discussion

**Figure 2:**
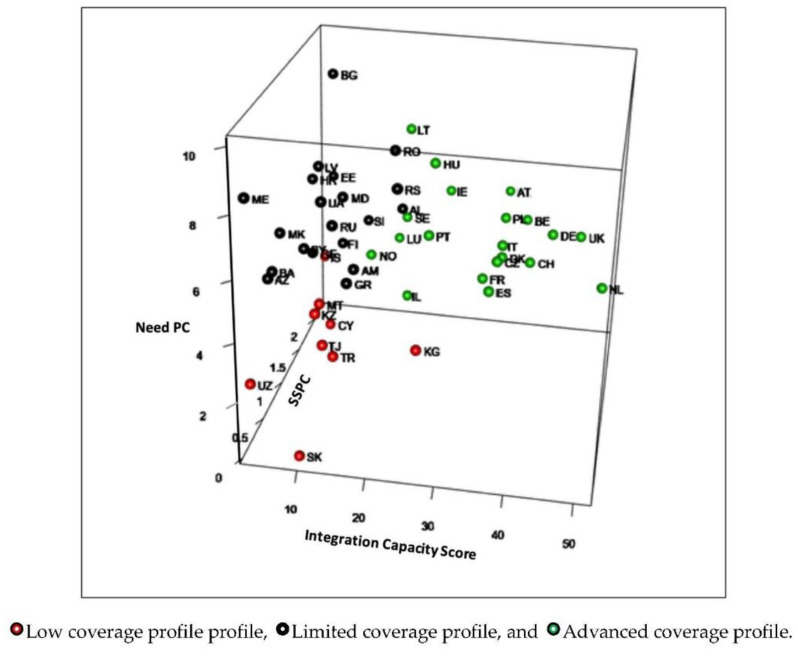
Cluster representation by country integration level.

On average, 0.7 ± 0.6 specialized palliative care services per 100,000 inhabitants were identified across European countries. The European Association for Palliative Care recommends two services per 100,000 inhabitants [21], suggesting that, on average, Europe offers nearly 75% less than the desirable number of specialized resources. Other available resources measured by the integration capacity score (compiling data on primary care, pediatric, cardiologic, oncologic, volunteer, and long-term care resources), also reached an insufficient mean score of 19.92/51. The prevalence of deaths due to SHS per 100,000 inhabitants showed an average of 5.6 ± 1.9, suggesting variable palliative care needs in their populations. These data explain that 59% of European countries provide limited or low coverage levels of palliative care, either because their palliative care needs are low (and so are the available resources), or because they have high needs but few specialized or non-specialized palliative care resources. The fact that countries with limited and low coverage levels demonstrated similar scores for the SSPC and the ICS, but different amounts of people needing palliative care, suggests that this was a determining factor. (Figure 2). Another factor that seems evident from the 2019 World Bank classification is that these countries seem to be lower-middle income: 14 countries (70%) had middle or low incomes [22]. Similarly, a legal framework to ensure access to palliative care is nearly inexistent, with scarce national palliative care strategies and regulations favoring palliative care integration [17].

This situation points to an urgent need for improvement. Countries at limited and low coverage levels, for instance, need to analyze the balance between specialized and integrated services and demand, since they show a borderline relationship regarding the capacity to guarantee care to patients needing palliative care. Their first actions should address the implementation of specialized services, strengthening primary and pediatric palliative care provision through existing resources, and initiating palliative care provision through other health disciplines (cardiology services), settings (long-term care facilities) and providers (volunteers) [2,23,24,25].

Measuring a country’s potential to provide palliative care to those in need through a single synthetic measure offers useful approaches to decision-making in public health, healthcare management, and the organization of healthcare services. It offers an improvement over earlier studies for estimating the coverage of palliative care across countries [12,15,26]. However, despite this, this measure features some limitations. For example, the disaggregated character of the primary indicators shows the situation for each indicator, but does not mean a dependent behavior of the other indicators, which require separate collection. This entails that indicators composing the final synthetic measure are many and imply a burden on data collection processes.

The advance towards synthetic indicators estimating palliative care provision regarding population needs is a future area of research. Future efforts should focus on studying variables for each dimension in depth in order to reduce the list of variables depicting the level of palliative care coverage, as well as adding other areas of integrated palliative care and quality of care.

## 5. Conclusions

The development of a synthetic indicator on the coverage of palliative care allows a comprehensive characterization of countries with advanced, limited, and low coverage levels. This facilitates decision-making and the design of public health programs to balance the relationship between supply and demand for palliative care in the region. Currently, 59% of European countries provide limited or low levels of palliative care through their national health systems.

## Figures and Tables

**Table 1:** Descriptive statistics for the variables: Integration Capacity Score (ICS), Specialized Services in Palliative Care per 100,000 inh. (SSPC), and Palliative Care Need.

Country	SSPC	ICS	Need PC
The Netherlands	0.9	51	4.7
UK	1.3	47	5.5
Germany	1.1	43	5.9
Switzerland	1.0	40	5.1
Belgium	1.7	38	5.1
Czech Rep.	0.6	36	5.9
Denmark	0.9	36	5.4
Italy	0.9	36	5.8
Poland	1.5	35	5.5
Spain	0.6	35	4.9
Austria	2.2	34	5.1
France	1.0	33	4.4
Kyrgyzstan	0.2	26	3.7
Hungary	1.1	25	7.9
Ireland	1.9	25	5.5
Portugal	0.9	25	5.9
Serbia	0.2	23	8.7
Albania	0.9	21	6.7
Romania	0.6	21	9.1
Israel	1.4	20	2.6
Lithuania	1.7	19	7.9
Sweden	1.6	19	5.0
Armenia	0.2	17	6.1
Greece	0.0	17	6.1
Luxembourg	1.8	17	3.8
Norway	1.2	15	4.4
Slovenia	1.1	15	5.8
Moldova	0.5	14	7.7
Russian Fed.	0.2	14	7.4
Turkey	0.2	14	3.2
Finland	0.7	13	5.8
Tajikistan	0.1	13	3.8
Ukraine	0.1	13	8.3
Kazakhstan	0.1	12	4.8
Malta	0.4	11	4.4
Belarus	0.2	10	6.6
Cyprus	0.9	10	2.5
Latvia	0.6	10	8.4
Slovakia	0.0	10	0.2
Georgia	0.6	9	5.6
Bulgaria	1.4	8	10
Croatia	0.8	8	7.6
Estonia	1.4	8	6.6
Azerbaijan	0.0	6	6.0
Bosnia & H.	0.1	6	6.0
Iceland	1.5	6	3.5
Macedonia	0.3	6	6.8
Montenegro	0.0	3	8.4
Uzbekistan	0.0	3	2.5
Mean	0.79	19.92	5.69
Std. Dev.	0.60	12.45	1.92
Kurtosis	−0.830	−0.397	0.494
Skewness	0.383	0.758	−0.197

**Table 2:** Response profile to SSPC, ICS and PC Need of the analyzed countries.

Country Coverage Level	Specialized PC Services		Integrated Resources in the Health System		PC Need		Countries
	N° Services per 100,000		Integration Capacity Score (ICS)		Deceased Patients with SHS per 100,000 Inhabitants, Year		*n* = 49
	Level	Mean (Range)	Level	Mean (Range)	Level	Mean (Range)	*n* (%)
Advanced Coverage profile	↑	1.27 (0.6–2.2)	↑	31.45 (15–51)	↓	5.32 (2.6–7.9)	20 (41)
Limited Coverage profile	↓	0.38 (0–1.5)	↓	11.67 (3–26)	↓↓	3.18 (0.2–4.8)	9 (18)
Low Coverage profile	↓	0.50 (0–1.4)	↓	12.10 (3–23)	↑↑	7.19 (5.6–10)	20 (41)

**Table 3:** Palliative care global development in European countries in comparison with the 2019 World Map levels.

Country Development Level	Country	World Map of Palliative Care 2019 Level ^1^
Advanced Coverage: countries with high scores on ICS, SSPC and low PC needs	The Netherlands	4b
	UK	4b
	Germany	4b
	Switzerland	4a
	Belgium	4b
	Czech Rep.	4a
	Denmark	4b
	Italy	4b
	Poland	4b
	Spain	4b
	Austria	4a
	France	4b
	Hungary	4a
	Ireland	4b
	Portugal	4b
	Israel	4b
	Lithuania	4b
	Sweden	4b
	Luxembourg	3b
	Norway	4b
Limited Coverage: countries with low levels of need and low ICS and SSPC scores	Kyrgyzstan	3b
	Turkey	3b
	Tajikistan	3b
	Kazakhstan	4a
	Malta	3b
	Cyprus	3b
	Slovakia	4a
	Iceland	4b
	Uzbekistan	2
Low Coverage: Countries with high levels of need and low levels of ICS and SSPC	Serbia	3b
	Albania	3b
	Romania	4b
	Armenia	3a
	Greece	3a
	Slovenia	3b
	Moldova	3a
	Russian Fed.	4a
	Finland	3b
	Ukraine	4a
	Belarus	3b
	Latvia	4a
	Georgia	4a
	Bulgaria	3b
	Croatia	3
	Estonia	3a
	Azerbaijan	3b
	Bosnia & H.	3a
	Macedonia	3b
	Montenegro	1

^1^.World map of palliative care levels: (1) no known palliative care activity; (2) capacity-building; (3a) isolated provision; (3b) generalized provision; (4a) preliminary integration into mainstream provision; (4b) advanced integration.

## Data Availability

Data management and sharing All EAPC Atlases data can be accessed from http://dadun.unav.edu/handle/10171/56787 (accessed on 12 October 2020) or requested from egarralda@unav.es.
